# Comparison of a Prototype for Indications-Based Prescribing With 2 Commercial Prescribing Systems

**DOI:** 10.1001/jamanetworkopen.2019.1514

**Published:** 2019-03-29

**Authors:** Pamela M. Garabedian, Adam Wright, Isabella Newbury, Lynn A. Volk, Alejandra Salazar, Mary G. Amato, Aaron W. Nathan, Katherine J. Forsythe, William L. Galanter, Kevin Kron, Sara Myers, Joanna Abraham, Sarah K. McCord, Tewodros Eguale, David W. Bates, Gordon D. Schiff

**Affiliations:** 1 Partners HealthCare System, Inc, Somerville, Massachusetts; 2 Harvard Medical School, Boston, Massachusetts; 3 Brigham and Women’s Hospital, Boston, Massachusetts; 4 Massachusetts College of Pharmacy and Health Sciences University, Boston; 5 Mayo Clinic, Rochester, Minnesota; 6 University of Illinois Hospital and Health Science System, Chicago; 7 Washington University School of Medicine in St Louis, St Louis, Missouri

## Abstract

**Importance:**

The indication (reason for use) for a medication is rarely included on prescriptions despite repeated recommendations to do so. One barrier has been the way existing electronic prescribing systems have been designed.

**Objective:**

To evaluate, in comparison with the prescribing modules of 2 leading electronic health record prescribing systems, the efficiency, error rate, and satisfaction with a new computerized provider order entry prototype for the outpatient setting that allows clinicians to initiate prescribing using the indication.

**Design, Setting, and Participants:**

This quality improvement study used usability tests requiring internal medicine physicians, residents, and physician assistants to enter prescriptions electronically, including indication, for 8 clinical scenarios. The tool order assignments were randomized and prescribers were asked to use the prototype for 4 of the scenarios and their usual system for the other 4. Time on task, number of clicks, and order details were captured. User satisfaction was measured using posttask ratings and a validated system usability scale. The study participants practiced in 2 health systems’ outpatient practices. Usability tests were conducted between April and October of 2017.

**Main Outcomes and Measures:**

Usability (efficiency, error rate, and satisfaction) of indications-based computerized provider order entry prototype vs the electronic prescribing interface of 2 electronic health record vendors.

**Results:**

Thirty-two participants (17 attending physicians, 13 residents, and 2 physician assistants) used the prototype to complete 256 usability test scenarios. The mean (SD) time on task was 1.78 (1.17) minutes. For the 20 participants who used vendor 1’s system, it took a mean (SD) of 3.37 (1.90) minutes to complete a prescription, and for the 12 participants using vendor 2’s system, it took a mean (SD) of 2.93 (1.52) minutes. Across all scenarios, when comparing number of clicks, for those participants using the prototype and vendor 1, there was a statistically significant difference from the mean (SD) number of clicks needed (18.39 [12.62] vs 46.50 [27.29]; difference, 28.11; 95% CI, 21.47-34.75; *P* < .001). For those using the prototype and vendor 2, there was also a statistically significant difference in number of clicks (20.10 [11.52] vs 38.25 [19.77]; difference, 18.14; 95% CI, 11.59-24.70; *P* < .001). A blinded review of the order details revealed medication errors (eg, drug-allergy interactions) in 38 of 128 prescribing sessions using a vendor system vs 7 of 128 with the prototype.

**Conclusions and Relevance:**

Reengineering prescribing to start with the drug indication allowed indications to be captured in an easy and useful way, which may be associated with saved time and effort, reduced medication errors, and increased clinician satisfaction.

## Key Points

**Question:**

Is a redesigned electronic prescribing workflow to better support the incorporation of the indication in the outpatient prescribing process associated with reduced errors and improved clinician experience?

**Findings:**

This quality improvement study compared an indications-based electronic prescribing prototype with that of 2 leading electronic health record vendors and found that the usability of the prototype system substantially outperformed both vendors’ prescribing systems in terms of efficiency, error rate, and satisfaction.

**Meaning:**

Reengineering prescribing to start with the drug indication allowed indications to be captured in an easy and useful way and may be associated with saved time and effort, reduced errors, and increased clinician satisfaction.

## Teaser

This quality improvement study compares efficiency, error rate, and user satisfaction of an indications-based electronic prescribing prototype vs the prescribing modules of 2 leading electronic health record vendors.

## Introduction

For nearly 4 decades, multiple medication safety organizations such as the National Coordinating Council for Medication Error Reporting and Prevention, the National Association of Boards of Pharmacy, and the National Council for Prescription Drug Programs have recommended including the indication (reason for the medication) on written prescriptions and medication bottle labels for safer and more understandable prescriptions.^1,2,3,4,5^ However, for a variety of reasons, progress has been slow in including the indication in medication orders.^6,7^ Based on a literature review and stakeholder input, we identified barriers preventing clinicians from including the drug indication on their outpatient electronic prescriptions, including lack of time for this added step, poor software design and integration of indications into ordering software and workflow, confidentiality concerns, and uncertainty of the value of this extra step.^7,8^ Nonetheless, when surveyed, physicians, pharmacists, and patients overwhelmingly favor inclusion of the indication in the prescription.^3,9^

To address this gap, we conducted a 4-year project funded by the Agency for Healthcare Research and Quality to help accelerate progress in incorporation of indications into prescribing. We found that in current systems there are a variety of ways to add indications to prescriptions, but these required clinicians to either manually enter free text or select various precoded indications. To avoid this extra step, we conceptualized a fundamentally different way of composing electronic prescriptions—one that could both capture the indication and also support clinicians in choosing the most appropriate drug. Properly designed, the clinician, rather than entering a drug, would instead enter an indication or choose one from the problem list. The reengineered computerized provider order entry (CPOE) system would suggest recommended treatment as well as capture indication. We hypothesized that this approach would make identifying and ordering the right drug faster, easier, and safer.

We designed a prescribing prototype following a user-centered design process involving contextual inquiry sessions, participatory design, usability roundtables, formative usability testing with think-aloud protocols, and iterative refinement of the prototype. We then conducted summative usability testing comparing the prototype to the prescribing interface of 2 leading commercial electronic health record (EHR) systems using 8 typical primary care prescribing scenarios. Our aim was to compare the efficiency, error rate, and satisfaction of the 3 systems using a mixed-methods qualitative and quantitative approach.

## Methods

This quality improvement study used usability testing to compare a prototype indications-based prescribing interface to that of 2 vendor EHRs. While it was not a quality improvement intervention, we followed the Standards for Quality Improvement Reporting Excellence (SQUIRE) reporting guideline when applicable.

### Prototype Description

**Figure 1.**
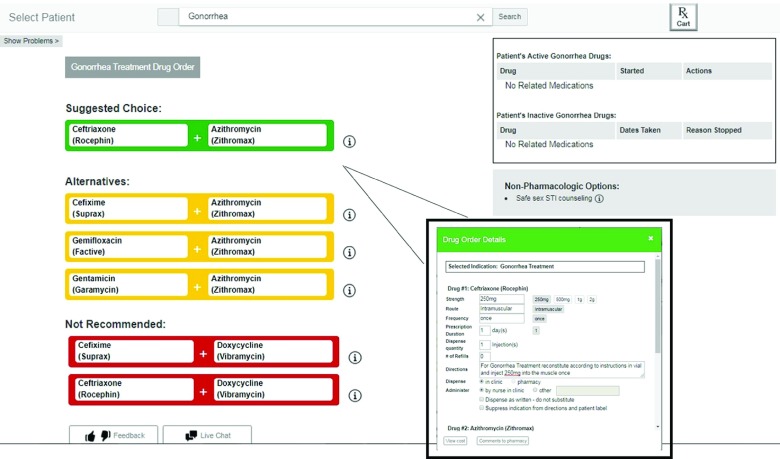
Screenshot From the Indications-Based Prescribing Prototype of the Gonorrhea Case Scenario A prescriber enters the indication in the search bar (or selects it from a preexisting problem list—not shown on the screen). The prototype then suggests drugs of choice with alternatives and drugs that are not recommended based on patient factors (eg, allergies), insurance formulary requirements, and evidence-based guidelines. After a drug is selected, the order details screen appears with most fields prepopulated with default options for dosing and frequency based on the indication and patient factors. Completing the order details adds the ordered drugs to the RxCart for final confirmation (next screen not shown here).

Following a period of user-centered design activities focused on understanding the value, barriers, desired features, and user requirements, we designed an indications-based prescribing CPOE prototype. It was designed with the option to enter a prescription by either searching for an indication or selecting a problem from the problem list. A key feature is the list of drugs of choice, or best-practice options, based on the selected indication. This patient-specific list of drug choices was customized for sample test patient scenarios based on evidence-based guidelines, US Food and Drug Administration labeled and nonlabeled indications, insurance formulary requirements, and patient-specific factors such as contraindications due to drugs, allergies or intolerance, or renal function. The medication options for any given indication were presented as suggested choice (in green), alternative (in yellow), or not recommended (in red) (Figure 1). The prototype also presented recommended initial dosing, frequency, and other order details based on the indication chosen and patient factors that would influence dosing, such as weight or renal function. Clinicians also had the option to search for and enter the drug directly (ie, as typical in CPOE). Immediately following a search for a specific drug, clinicians were required to select an indication from a list of drug-specific indications to associate with the order. Our prototype automatically displayed the indication on the prescription instructions, except for potentially sensitive conditions such as psychiatric diagnoses or sexually transmitted infections, for which the default was to suppress the indication. We allowed clinicians to change the default choice of including the indication in the instructions by presenting it as a check box on the order details screen (eFigure in the Supplement). The prototype was designed to support multidrug treatment regimens and multiple indications if warranted.

### Usability Testing

#### Recruitment

Eligible participants included internal medicine faculty, residents, and physician assistants who were using either Epic or Cerner systems to prescribe electronically in the outpatient setting. A convenience sample of eligible participants from Partners HealthCare and University of Illinois Hospital were emailed a recruitment letter and frequently asked questions document by the principal investigator or site investigator. Informed consent was obtained verbally prior to the usability test. This study was approved by the Partners HealthCare and the University of Illinois at Chicago human subjects research committees. Usability tests were conducted between April and October of 2017.

#### Clinical Scenarios

Eight clinical scenarios were developed by the project pharmacists (A.S. and M.G.A.) in collaboration with additional clinicians and subject matter experts on our research team (A.W., T.E., D.W.B., G.D.S., and W.L.G.) (eTables 1-8 in the Supplement). Medication recommendation choices were identified by review of practice guidelines for each clinical condition in the 8 scenarios. The scenarios included a combination of common primary care ambulatory problems, such as poorly controlled hypertension, migraine prophylaxis, gout flare, and newly diagnosed diabetes. In addition, 2 scenarios, gonorrhea and *Helicobacter pylori* infection, required multimedication combinations with specific dosing schedules and treatment durations. Each scenario was designed to consider various challenges that clinicians often encounter at the time of prescribing, such as patient-specific factors that affect treatment choices (allergies, renal impairment, and comorbidities). We also included 2 scenarios designed to provoke common look-alike or sound-alike medication errors: hydroxyzine vs hydralazine and risperidone vs ropinirole.^10^ To make the medication ordering as realistic as possible, case scenarios included relevant clinical data (eg, renal and liver function panels and glycated hemoglobin levels) that may influence the drug options that would be appropriate for the patient. Test patients were created in the prototype system and each vendor system so that the medical records matched the clinical information supporting the scenarios.

#### Test Procedures

Each hour-long test session was held either in a clinical office or conference room. We deployed Morae usability software version 3.3.4 (Techsmith) on a laptop with a wireless mouse to record the sessions.^11^ An observer and note-taker (I.N.) attended each session along with the moderator (P.M.G.). Participants completed 4 scenarios with the prototype system and 4 scenarios with their usual commercial EHR. Participants were asked to complete the following task for each scenario: “Review the patient’s history and order the appropriate medication(s) for this patient. Please include the indication on the prescription for the pharmacist and patient (unless you have concerns about sensitive information).” We randomly alternated the CPOE system and the 4 scenarios assigned to participants to avoid ordering and sequential difficulty effects. Participants were provided with a 2-minute demonstration of the prototype system before beginning the tasks and were instructed to use their vendor system as they normally would. Participants were provided with no additional requirements or instruction in either the prototype system or the vendor system on using any specific method to include the indication. At study completion, participants received a $50 gift card.

#### Usability Metrics

To capture time on task for each scenario, the moderator selected the appropriate patient record in the system and brought the participant to a consistent starting point in both the prototype and vendor systems, although participants could navigate away from this screen once they began. The start time for each task began when the participant took the mouse and began to navigate. The task ended when the participant successfully completed the task or informed us that they were done with the orders for the scenario.

We recorded medications prescribed during each scenario and calculated an error rate based on an independent review of each order by 2 pharmacists. In cases in which the pharmacists disagreed, this was adjudicated by an internal medicine physician. The pharmacists and physician were blinded to which system was used when determining whether the medication order was an error. We also recorded whether the participants added the indication to the prescription for each scenario. In the prototype, the indication was automatically captured when initiating the order, whereas in the vendor systems, the participant could either enter it using free text in the patient prescription instructions or check prespecified fields available in the vendor system. We did not include the gonorrhea scenario in this analysis owing to the sensitive nature of the condition. We also recorded whether participants used outside reference resources for additional information during each scenario.

The single ease question (SEQ) was administered at the completion of each scenario.^12^ Participants were asked to respond to the question, “Overall, how difficult or easy was this task to complete?” on a scale from 1 to 7 with 1 indicating it was very easy and 7 indicating it was very difficult. Average SEQ ratings were calculated using data from participants who used the same CPOE system (prototype, vendor 1, or vendor 2) to complete a given scenario. Participants completed an overall system usability scale for the prototype at the end of the test session.^13,14^ The total system usability scale was averaged across all participants.

### Statistical Analysis

Data analysis was done using SAS statistical software version 9.3 (SAS Institute).^15^ We used a Mann-Whitney *U* test to compare the prototype to each vendor system on efficiency and ease of use for each individual scenario. We did a pooled analysis using *t* tests to compare the overall mean time on task, clicks, and SEQ rating for the prototype and each vendor system across all scenarios. We used χ^2^ tests to analyze the proportion data on access to outside reference source and prescribing errors.^16^ A 2-sided *P* value less than .05 was considered statistically significant.

## Results

Overall, 17 faculty, 13 residents, and 2 physician assistants completed the usability tests, entering medications for a total of 256 test scenarios.^17,18^ All 32 participants used the prototype system; of this group 20 completed tasks using vendor 1’s system and 12 used vendor 2’s system. Most participants had more than 2 years of experience with their current EHR system and 82% reported having an intermediate or higher level of skill with technology (Table 1).

**Table 1.** Characteristics of 32 Participants

Characteristic	No. (%)
Clinical role	
Attending physician	17 (53)
Physician assistant	2 (6)
Resident (second, third, or fourth year)	13 (41)
Time using current system, y	
<2	5 (16)
2-4	17 (53)
5-10	6 (19)
>10	4 (13)
Level of skill with technology	
Novice	1 (3)
Novice-intermediate	2 (6)
Intermediate	15 (47)
Intermediate-expert	5 (16)
Expert	9 (28)
Do you use indications with Epic or Cerner now?	
Yes, link to diagnosis	7 (22)
For specific reasons, but not everything	8 (25)
Sometimes	7 (22)
No	10 (31)

### Efficiency

**Figure 2.**
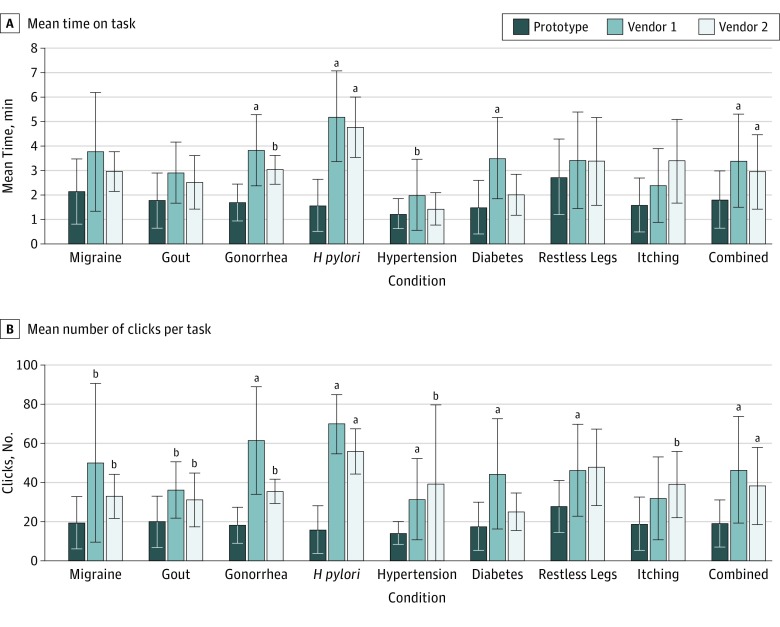
Usability Test Results of Time on Task and Clicks Results of the usability testing on the prototype (32 participants), vendor 1 (20 participants), and vendor 2 (12 participants) are shown for time on task and number of clicks. Although the prototype measure shown is that for all participants, for statistical tests the participants who used vendor 1 were compared with their performance on the prototype, as also done with vendor 2. *H pylori* indicates *Helicobacter pylori*; error bars, 95% confidence intervals. ^a^*P* < .05. ^b^*P* < .01.

Across all 32 participants, the mean (SD) time on task to complete a medication order using the prototype was 1.78 (1.17) minutes. Participants using vendor 1 had a mean (SD) time on task of 3.37 (1.90) minutes and those using vendor 2 had a mean (SD) time on task of 2.93 (1.52) minutes. When comparing the participants who used both the prototype and vendor 1, ordering with the prototype was significantly faster for 4 of the scenarios. For the participants who used vendor 2, the prototype was significantly faster for 2 scenarios (Figure 2). When we pooled the data across all the scenarios, the mean time for the prototype was significantly faster than either of the vendor systems (Figure 2).

The mean (SD) number of clicks to complete a scenario in the prototype was 19.0 (12.2). For those using the prototype and vendor 1, there was a statistically significant difference from the mean (SD) number of clicks needed pooled across all scenarios (18.39 [12.62] vs 46.50 [27.29]; difference, 28.11; 95% CI, 21.47-34.75; *P* < .001). For those using the prototype and vendor 2, there was also a statistically significant difference in number of clicks across scenarios (20.10 [11.52] vs. 38.25 [19.77]; difference, 18.14; 95% CI, 11.59-24.70; *P* < .001). The difference in the number of clicks for those participants using both the prototype and vendor 1 was statistically significant for all scenarios but itching. There was a statistically significant difference in the number of clicks when comparing the prototype with vendor 2 for 6 of the 8 scenarios (Figure 2).

**Figure 3.**
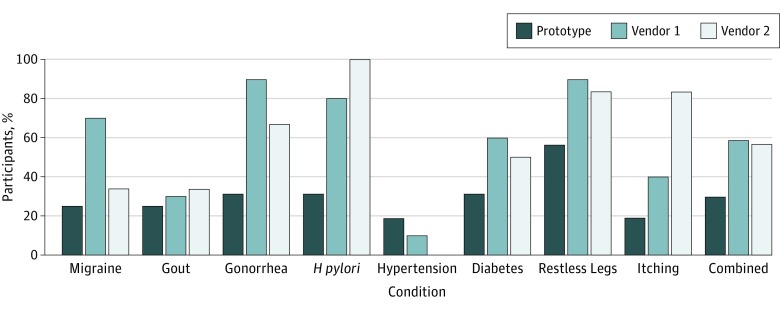
Usability Test Results of Access to Outside Reference Source The percentage of participants who accessed an outside reference source during the ordering tasks is shown for each diagnosis for the prototype, vendor 1, and vendor 2. *H pylori* indicates *Helicobacter pylori.*

Overall, for the 8 scenarios, 28.8% of participants (23 of 80) accessed an outside reference source for additional information during the ordering task when using the prototype and 58.8% of participants (47 of 80) accessed an outside reference when using vendor 1 (difference, 30.00%; 95% CI, 15.35%-44.65%; *P* < .001). Moreover, there was also a statistically significant difference between the participants who sought an outside reference source using the prototype vs vendor 2 (31.3% [15 of 48] vs 56.3% [27 of 48]; difference, 25.00%; 95% CI, 5.79%-44.21%; *P* = .01) (Figure 3).

### Error Rate

Across all participants, 7 of 128 (5.5%) prescribing orders using the prototype were classified as errors compared with 38 of 128 (29.7%) with a vendor system (difference, 24.22%; 95% CI, 15.38%-33.06%; *P* < .001). Look-alike or sound-alike errors occurred in 0.8% [1 of 128] of orders in the prototype, 2.5% [2 of 80] in vendor 1, and 2.1% [1 of 48] in vendor 2; these differences were not statistically significant. Reasons that an order was considered erroneous included an incorrect route, frequency, duration, or dose as well as instances in which part of a multidrug treatment was missing (eTable 9 in the Supplement). As designed, the prototype automatically included the indication on the order 100% of the time, excluding the gonorrhea scenario. Orders entered electronically using vendor 1’s CPOE included the indication 61% of the time, while those entered using vendor 2’s CPOE included the indication 62% of the time.

### Satisfaction

The mean (SD) response to the SEQ was 1.73 (0.97) across all scenarios on the prototype, while the mean (SD) rating was 3.70 (1.77) for vendor 1 and 2.75 (1.39) for vendor 2. For the participants who used the prototype and vendor 1, the SEQ rating for the prototype was higher with a statistically significant result for all but 1 scenario, itching. For the participants who used the prototype and vendor 2, the prototype was more highly rated, although only the *H pylori* scenario rating showed a statistically significant result (Table 2). The mean (SD) system usability scale score for the prototype across all participants in the study was 89.7 (9.37), which is rated to be excellent in comparison with a wide range of other applications.^19^

**Table 2.** Responses to Single Ease Question for Each Scenario^a^

Scenario	Mean (SD)			
	Site 1 (n = 20)		Site 2 (n = 12)	
	Prototype	Vendor 1	Prototype	Vendor 2
Migraine	1.80 (0.79)	3.90 (1.45)^b^	2.00 (1.10)	2.50 (0.84)
Gout	1.90 (1.20)	3.50 (1.84)^c^	1.50 (0.55)	2.83 (1.83)
Gonorrhea	1.30 (0.67)	4.10 (1.66)^b^	2.00 (0.89)	2.83 (0.98)
*Helicobacter pylori*	1.80 (1.03)	4.60 (1.58)^b^	1.33 (0.82)	3.83 (1.60)^b^
Hypertension	1.10 (0.32)	2.50 (1.27)^b^	1.67 (1.21)	2.17 (0.75)
Diabetes	1.50 (0.53)	3.90 (2.08)^b^	1.50 (0.84)	2.17 (1.33)
Restless legs	1.70 (1.06)	3.50 (1.84)^b^	2.67 (1.37)	2.67 (0.82)
Itching	2.00 (0.82)	3.60 (2.12)	2.33 (1.75)	3.00 (2.28)
Combined	1.64 (0.84)	3.70 (1.77)^b^	1.88 (1.12)	2.75 (1.39)^b^

^a^ Participants were asked after each task, “Overall, how difficult or easy was the task to complete?” Responses were on a 7-point rating scale with 1 indicating that the task was very easy and 7 indicating that it was very difficult.

^b^ Significant at *P* < .01.

^c^ Significant at *P* < .05.

## Discussion

Despite the steady increase in use of EHRs in the United States, clinicians remain frustrated with many aspects of design and workflow, including medication ordering and medication-related decision supports.^20,21,22,23^ In addition, current systems do little to facilitate adding indications into prescription orders, something that has been recommended by pharmacists and safety experts for several decades.^1,2,4,5^ Working with very modest resources during a 1-year period of usability research, prototyping, and development, we designed an innovative CPOE prototype that changes the way prescribers order drugs by offering the option of starting with the indication, thereby permitting the computer to suggest optimal drug regimen choices. We found this approach outperformed 2 leading vendor systems’ electronic prescribing modules on all scenarios in aggregate in terms of efficiency, error reduction, and satisfaction. Despite minimal training on the prototype, participants completed tasks in roughly half the time and entry clicks when ordering with the prototype compared with both vendor systems. These time savings have major clinical implications, as ordering a medication is one of the most frequent tasks performed in clinical encounters. In addition, orders generated with the prototype included the indication 100% of the time (excluding the gonorrhea scenario) and had fewer medication errors than those placed in the vendor systems.

The prototype offers several interface design features in alignment with human factors and usability principles that help explain our findings. To better align with a clinician’s thought process, the prototype was organized with a focus on problem-based workflow. Providing a list of drugs of choice that proactively considered and incorporated patient-specific factors, allergies, and contraindications eased the cognitive burden on the ordering clinician and may have major implications for decreasing after-the-fact CPOE alerts and resulting alert fatigue. Clinicians could use their working memory, concentrating on other aspects of care (such as shared decision making with the patient to confirm or select the appropriate medication choice) rather than having to recall medication names and doses or searching for or remembering details of a patient’s history. While some clinicians might be expected to prefer to simply enter the drug as they usually would and resist a shift in their electronic ordering workflow, our study found that with minimal training on the prototype (2 minutes, compared with years of experience with their familiar EHR’s prescribing interface), all clinicians quickly learned, adopted, and overwhelmingly preferred this new workflow. This occurred despite the fact that the prototype was designed to preserve clinician autonomy and flexibility to deviate from the recommended suggestions.

One noteworthy finding was the frequency with which participants accessed outside reference materials with their usual ordering systems, and how dramatically this decreased with the prototype system. In particular, gonorrhea and *H pylori* would more often require a physician to access outside reference material for information on drugs and dosing owing to more complicated multidrug combination therapy and evolving antibiotic resistance patterns; our system significantly reduces this time burden in these situations. In the prototype, each of the suggested drug choices included information (that the prescriber viewed by hovering over the choice) explaining the rationale for that recommendation. Only rarely, when participants questioned whether they could trust the offered prototype recommendations, did they choose to check other reference sources.

The primary motivation for our redesigned ordering system was the need to more efficiently and effectively capture and display the indication for the prescription.^24,25^ By doing so, the indications could be both communicated to the pharmacist and likely placed on the patient’s medication bottle label. Doing so could help with patient and caregiver understanding of the medication and thereby potentially improve medication safety through preventing medication mix-ups and aiding adherence.^7,8,26^ Currently, the indication is rarely entered in the patient instructions or displayed on the patient’s medication bottle, despite strong recommendations to do so and a general agreement by clinicians that this would be desirable and beneficial (except in sensitive situations such as sexually transmitted infections or mental health diagnoses).

Interviews with clinicians following the usability tests confirmed several features and concerns we had collected in earlier design phases of this project.^8^ These issues and suggestions centered around integration with the rest of the EHR and the quality and reliability of back-end knowledge required for the drug or regimen choice recommendations. Differentiating an indication from a billing diagnosis is a related issue that requires further conceptual and design considerations.^8^ In addition to defining and maintaining the indication drug database, there are other technical and policy issues that were raised, including challenges surrounding the generation of drug recommendations based on patient factors, transmitting the indication information to pharmacy systems, and mapping indications to patient-friendly terms appropriate for patient medication labels and accompanying leaflets.

Generally, EHR vendors have shied away from risking legal liability by incorporating treatment recommendations into their systems. However, several clinical content vendors have begun to create databases linking indications with drugs, and we have been encouraged by favorable reception of the prototype by a wide variety of participants in the drug utilization and informatics arena and have been in discussion with EHR vendors about ways such a revamped method of ordering could be incorporated into their CPOE systems. This will critically depend on production of evidence-based, trustworthy guidelines and guidance to populate recommended drug choices.

### Limitations

This study has limitations. The prototype was built around only 8 scenarios. We chose scenarios to test a broad range of typical and frequently occurring primary care prescribing issues. However, we recognize that additional usability issues will inevitably arise when we increase the scope and complexity of the indications and medications. Also, the fidelity of the fully functioning vendor EHRs and our prototype CPOE system was not a direct comparison, although we attempted to ensure that all clinical data available in the record and the point of access were the same regardless of the system. While the pharmacists who independently reviewed the safety and appropriateness of the orders were blinded to which system was used to generate them, the participants and observers obviously were not blinded. This could have introduced bias in favor of the test system, although the scenarios and request for including indication were based on evidence guidelines and recommendations to include indications. Participants had limited training on our system vs longer lengths of training (lengths varied, but were usually several years or more) on their usual vendor system, which should have advantaged their speed and comfort with their vendor system.

Testing was performed in a simulated test environment. For this type of system to be successful, maintaining other clinical data that inform the decision support is required, such as the problem list. Our hope is that this redesigned workflow could support better integration of the problem list and ordering system. Furthermore, our system included a feature to permit adding the problem related to the indication to the problem list with a single click, which has been shown to improve problem list maintenance.^27^

## Conclusions

We found that a prototype built around electronically prescribing medications by indication outperformed the electronic prescribing modules of 2 vendor EHR systems in terms of improving efficiency and satisfaction and reducing errors in aggregate across 8 clinical scenarios. These results suggest that there is substantial room for improvement in current applications, and that adopting this type of approach could improve both prescriber efficiency and reduce errors, while giving patients key information that may help with medication safety and adherence.

## Notes

Supplement. **eFigure.** Indications-Based Electronic Prescribing Prototype Migraine Test Scenario Screen Capture With Highlighted Features **eTable 1.** Migraine Scenario and Test Patient Data **eTable 2.** Gout Scenario and Test Patient Data **eTable 3.** Gonorrhea Scenario and Test Patient Data **eTable 4.** *H pylori* Scenario and Test Patient Data **eTable 5.** Hypertension Scenario and Test Patient Data **eTable 6.** Diabetes Scenario and Test Patient Data **eTable 7.** Restless Leg Syndrome Scenario and Test Patient Data **eTable 8.** Chronic Idiopathic Urticaria (“Itching”) and Test Patient Data **eTable 9.** Type and Number of Prescribing Errors When Using the Prototype and Vendors During Usability Testing
