# Supplementary material for: Comparison of a Prototype for Indications-Based Prescribing With 2 Commercial Prescribing Systems
Source: JAMA Netw Open. 2019 Mar 29;2(3):e191514. doi: 10.1001/jamanetworkopen.2019.1514 (PMC6450312; doi:10.1001/jamanetworkopen.2019.1514)
Supplement: Supplement. — eFigure. Indications-Based Electronic Prescribing Prototype Migraine Test Scenario Screen Capture With Highlighted Features eTable 1. Migraine Scenario and Test Patient Data eTable 2. Gout Scenario and Test Patient Data eTable 3. Gonorrhea Scenario and Test Patient Data eTable 4. H pylori Scenario and Test Patient Data eTable 5. Hypertension Scenario and Test Patient Data eTable 6. Diabetes Scenario and Test Patient Data eTable 7. Restless Leg Syndrome Scenario and Test Patient Data eTable 8. Chronic Idiopathic Urticaria (“Itching”) and Test Patient Data eTable 9. Type and Number of Prescribing Errors When Using the Prototype and Vendors During Usability Testing [file jamanetwopen-2-e191514-s001.pdf]

Supplementary Online Content

Garabedian PM, Wright A, Newbury I, et al. Comparison of a prototype for indications-based prescribing with 2 commercial prescribing systems. *JAMA Netw Open*. 2019;2(3):e191514. doi:10.1001/jamanetworkopen.2019.1514

eFigure. Indications-Based Electronic Prescribing Prototype Migraine Test Scenario Screen Capture With Highlighted Features

eTable 1. Migraine Scenario and Test Patient Data

eTable 2. Gout Scenario and Test Patient Data

eTable 3. Gonorrhea Scenario and Test Patient Data

eTable 4. *H pylori* Scenario and Test Patient Data

eTable 5. Hypertension Scenario and Test Patient Data

eTable 6. Diabetes Scenario and Test Patient Data

eTable 7. Restless Leg Syndrome Scenario and Test Patient Data

eTable 8. Chronic Idiopathic Urticaria (“Itching”) and Test Patient Data

eTable 9. Type and Number of Prescribing Errors When Using the Prototype and Vendors During Usability Testing

This supplementary material has been provided by the authors to give readers additional information about their work.

© 2019 Garabedian PM et al. *JAMA Network Open.*

eFigure: Indications-based electronic prescribing prototype migraine test scenario screen capture with highlighted features

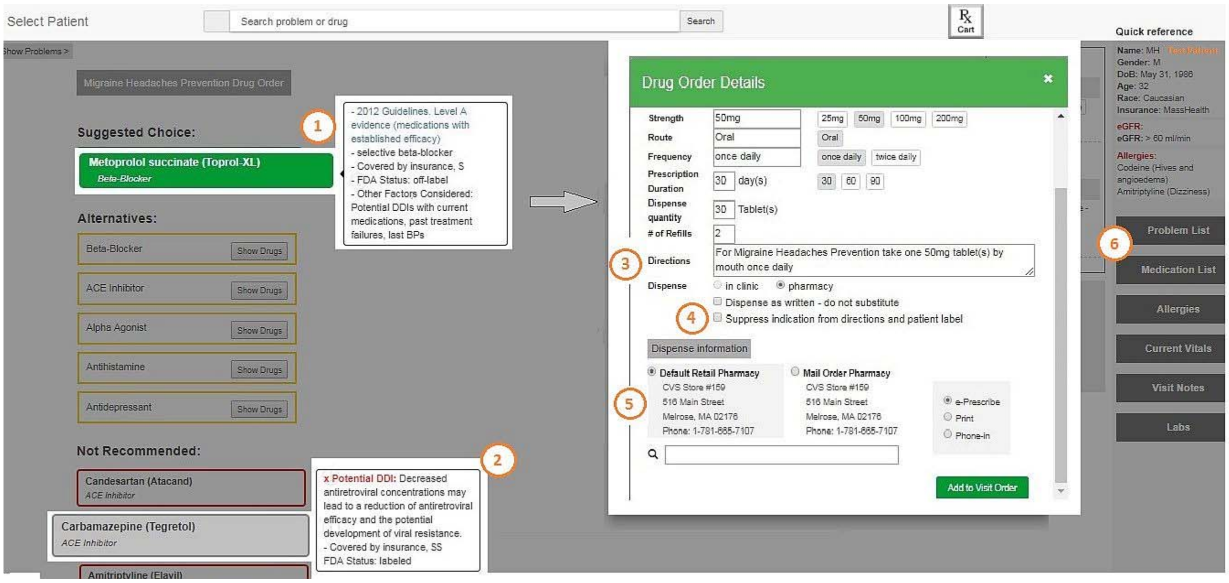

eFigure Legend *1.* After selecting 'migraine' from the left-hand side patient active problem list menu (not shown here), the clinician can view all the drug options for that indication and hover-over any of them to see the patient specific factors, evidence-based guidelines, insurance formulary requirements and FDA status information that were used in classifying this drug as either a suggested choice, alternative, or not recommended drug for this patient and indication. *2.* This example highlights a 'not recommended' drug and the hover-over information regarding the drug-drug interaction that classified it as such. This patient has HIV and is being treated for it with anti-retroviral drugs making the drug carbamazepine contraindicated. *3.* Once the clinician selects the drug, the order details screen appears. The details are pre-populated with defaults based on the indication and patient factors, such as renal status, weight and age. They can be changed using the provided buttons or free text. The Directions field automatically updates when the other fields are changed and can be edited directly. *4.* There is a checkbox that allows the clinician to suppress the indication from the directions and the patient label in cases where the clinician or patient does not want it presented. By default, the checkbox would be "checked' for potentially sensitive conditions such as psychiatric or sexually transmitted infections. Otherwise, the indication would be automatically included with the directions and patient label. *5.* Other features include the ability to select the pharmacy and additional dispensing information. *6.* The right-side menu includes quick access to the patient's demographics, allergies, renal status, problem list, medication list, recent vitals, lab results, and visit notes.

© 2019 Garabedian PM et al. *JAMA Network Open.*

eTable 1: Migraine Scenario and Test Patient Data

Scenario/Testing Objectives:	Indication: Migraine Prevention<br>• Support selection of drug for prevention or treatment purposes<br>• Exclude contraindicated/previously failed drugs from recommended drugs<br>• Identify contraindications relating to existing patient problems
Scenario Prompt:	MH, a 30-year old male, comes to you complaining of recurrent migraines. He currently takes Naproxen to help treat his symptoms but is looking for something else to prevent his headaches. His blood pressure today was 138/83.
Patient Name:	MH
Gender:	Male
DOB:	5/31/1986
Age:	30
Race:	Caucasian
Insurance:	Mass Health
Medication List:	• Naproxen tablet 220mg: 1 po bid PRN headaches<br>• Ritonavir tablet 100mg 1 po qd<br>• Darunavir tablet 800mg 1 po qd<br>• Emtricitabine/Tenofovir tablet 200/300mg 1 po qd<br>• Inactive/Past Med:<br>o Amitriptyline (12/1/2014 - 1/1/2015) Caused dizziness
Problem List:	• Migraine Headaches<br>• HIV Disease<br>• Insomnia<br>*Inactive Problems*<br>• Gonorrhea<br>• Cough<br>• Acne
Recent Labs:<br>(DATE for all labs can be 4/2/17)	• Renal panel:<br>o SCr: 0.86 (range: 0.5-1.1); eGFR > 60 ml/min; Albumin 4.5 (3.5-5 G/DL), Calcium 9.5 (8-10.5mg/DL), CO2 28 (19-28 mmol/L), chloride 106 (98-110mmol/L), glucose 75 (70-100mg/DL), Potassium 3.9 (3.1-5.3 mmol/L), Sodium 143 (135-145mmol/L); Urea Nitrogen –BUN- 10 (7-25mg/DL)<br>• Hepatic function panel:<br>o albumin 4.2 (3.5-5 G/DL); bilirubin total 1.1 (0.3-1.2 MG/DL); bilirubin direct 0.3 (0-0.4mg/DL); alkaline phosphatase total 71 (25-100 U/L); Protein total 7.4 (6.8-8.6 g/DL) ALT: 23 (range: 9-67 U/L), AST: 29 (range: 13-39 U/L)
Recent Vitals:	• Weight: 145 lbs (as of 4/2/2017)<br>• Height: 5 ft 10 inches<br>• BMI: 20.8<br>• Last 3 Blood Pressure: 135/85 (4/2/2017); 130/82 (4/2/2017); 138/83 (as of 4/2/2017)<br>• Heart Rate: 88 (as of 4/2/2017)
Allergies/Drug Reactions:	• Codeine (hives and angioedema)<br>• Amitriptyline (Caused dizziness, he tried for Migraines from 12/1/2014 - 1/1/2015)

© 2019 Garabedian PM et al. *JAMA Network Open.*

eTable 2: Gout Scenario and Test Patient Data

Scenario/Testing Objectives:	Indication: Acute gout<br>• Test the inclusion of taper medications (steroid taper)<br>• Support selection of drug for prevention of gout attack vs treatment of gout attack<br>• Incorporate newly published guidelines (includes prednisone and NSAIDS as first choice in addition to Colchicine) in the suggested drug list
Scenario Prompt	A 55 year old male patient, GT, comes in to see you because he is having an acute gout attack. Upon physical exam, the patient has moderate pain affecting the large joint in the big toe.
Patient Name:	GT
Gender:	Male
DOB:	2/13/1962
Age:	55
Race:	Hispanic
Insurance:	Mass Health
Medication List:	• Docusate 100mg capsule: 1 capsule 2 times daily
Problem List:	• Constipation
Recent Labs:<br>(DATE for all labs can be 4/2/17)	• Renal panel:<br>o SCr: 1.2 (range: 0.5-1.1), eGFR >60ml/min, Albumin 4 (3.5-5 G/DL), Calcium 10 (8-10.5mg/DL), CO2 24 (19-28 mmol/L), chloride 109 (98-110mmol/L), glucose 185 (70-100mg/DL), Potassium 4 (3.1-5.3 mmol/L), Sodium 140 (135-145mmol/L); Urea Nitrogen –BUN- 28 (7-25mg/DL);<br>• Hepatic function panel:<br>o albumin 5 (3.5-5 G/DL); bilirubin total 1.1 (0.3-1.2 MG/DL); bilirubin direct 0.3 (0-0.4mg/DL); alkaline phosphatase total 50 (25-100 U/L); Protein total 7 (6.8-8.6 g/DL); ALT: 20 (range: 9-67 U/L), AST: 18 (range: 13-39 U/L)
Recent Vitals:	• Weight: 205 lbs (as of 4/2/2017)<br>• Height: 5 ft 10 inches<br>• BMI: 29.4<br>• Blood Pressure (last 3): 118/88 (4/2/2017); 120/82 (4/2/2017); 130/85 (as of 4/2/2017)
Allergies/Drug Reactions:	• Sulfamethoxazole and Trimethoprim (Unknown reaction)

© 2019 Garabedian PM et al. *JAMA Network Open.*

eTable 3: Gonorrhea Scenario and Test Patient Data

Scenario/Testing Objectives:	Indication: Gonorrhea<br>• Presentation of multiple drug combinations<br>• Impact of periodically changing guidelines, which are due to changing resistance patterns, on suggested drug list<br>• Referencing an authoritative source for guidelines (CDC) to determine suggested drugs<br>• Support for drugs Infrequently prescribed
Scenario Prompt:	A 19 year old heterosexual male patient, GC, comes in the office with symptoms of uncomplicated Gonorrhea and penile purulent discharge. This is the first time the patient has been diagnosed with Gonorrhea.
Patient Name:	GC
Gender:	Male
DOB:	6/29/1997
Age:	19
Race:	Caucasian
Insurance:	Mass Health
Medication List:	• Isotretinoin 20mg 1 po qd
Problem List:	• Acne<br>*Inactive Problems*<br>• Insomnia
Recent Labs:<br>(DATE for all labs can be 4/2/17)	• Ordered/Pending:<br>o RPR: non-reactive<br>o Chlamydia trachomantis antigen result: negative<br>o Neisseria gonorrhoeae antigen: positive<br>• Renal panel:<br>o SCr: 0.5 (range: 0.5-1.1); eGFR > 60 ml/min; Albumin 5 (3.5-5 G/DL), Calcium 8.5 (8-10.5mg/DL), CO2 15 (19-28 mmol/L), chloride 100 (98-110mmol/L), glucose 90 (70-100mg/DL), Potassium 4 (3.1-5.3 mmol/L), Sodium 143 (135-145mmol/L); Urea Nitrogen –BUN- 10 (7-25mg/DL)<br>• Hepatic function panel:<br>o albumin 4 (3.5-5 G/DL); bilirubin total 1.2 (0.3-1.2 MG/DL); bilirubin direct 0.4 (0-0.4mg/DL); alkaline phosphatase total 95 (25-100 U/L); Protein total 7.5 (6.8-8.6 g/DL); ALT: 70 (range: 9-67 U/L), AST: 38 (range: 13-39 U/L)
Recent Vitals:	• Weight: 190 lbs (as of 4/2/2017)<br>• Height: 5 ft 10 inches<br>• BMI: 27.3<br>• Blood Pressure: 121/82 mm Hg (as of 4/2/2017)
Allergies/Drug Reactions:	• Sulfamethoxazole and Trimethoprim (Rash when he was 5 y/o)

© 2019 Garabedian PM et al. *JAMA Network Open.*

eTable 4: *H. pylori* Scenario and Test Patient Data

Scenario/Testing Objectives:	Indication: *H. pylori*<br>• Presentation of multiple drug combinations<br>• Support for drugs Infrequently prescribed<br>• Incorporating periodically changing guidelines, which are due to changing resistance patterns<br>• Identify challenges of considering existing allergy to penicillin (which would be first recommended option)<br>• Incorporate challenge of insurance limitations regarding multi-drug pre-packets (treatment pack called Prevpac w/amoxicillin).
Scenario Prompt:	A 42-year old female patient, HP, comes in with *H. pylori* that was identified on upper endoscopy. There is low *H. pylori* resistance in the community and she is treatment naive.
Patient Name:	HP
Gender:	Female
DOB:	2/13/1975
Age:	42
Race:	Unknown
Insurance:	Mass Health
Medication List:	• Hydrochlorothiazide 25mg: 1 po qd
Problem List:	• Hypertension<br>• Cough<br>• Acne
Recent Labs:<br>(DATE for all labs can be 4/2/17)	• Renal panel:<br>o SCr: 1.2 (range: 0.5-1.1); eGFR > 60 ml/min; Albumin 3.8 (3.5-5 G/DL), Calcium 10 (8-10.5mg/DL), CO2 20 (19-28 mmol/L), chloride 99 (98-110mmol/L), glucose 90 (70-100mg/DL), Potassium 5 (3.1-5.3 mmol/L), Sodium 140 (135-145mmol/L); Urea Nitrogen –BUN- 8 (7-25mg/DL)<br>• Hepatic function panel:<br>o albumin 5 (3.5-5 G/DL); bilirubin total 1.1 (0.3-1.2 MG/DL); bilirubin direct 0.3 (0-0.4mg/DL); alkaline phosphatase total 50 (25-100 U/L); Protein total 7 (6.8-8.6 g/DL); ALT: 10 (range: 9-67 U/L), AST: 15 (range: 13-39 U/L)
Recent Vitals:	• Weight: 170 lbs (as of 4/2/2017)<br>• Height: 5 ft 5 inches<br>• BMI:28.3<br>• Blood Pressure: 140/90 mm Hg (as of 4/2/2017)
Allergies/Drug Reactions:	• Ampicillin/Sulbactam (Unasyn) (SOB & angiodema)<br>• Aspirin: hives and SOB

© 2019 Garabedian PM et al. *JAMA Network Open.*

eTable 5: Hypertension Scenario and Test Patient Data

Scenario/Testing Objectives:	Indication: Hypertension<br>• Test utility for common/frequent primary care problem<br>• Identify patient co-morbidities that influence drug suggestions<br>• Presenting multiple drug classes for alternatives<br>• Incorporating recent changes to guidelines<br>• Presenting many drug choices<br>• Consider ethnicity factor in drug suggestions<br>• Incorporate previously failed treatment factor in drug suggestions<br>• Challenge of poorly controlled – helping prescribers with next choice
Scenario Prompt:	A new patient, HT, is a 48 year old African American man with poorly controlled blood pressure on Hydrochlorothiazide. His blood pressure today was 170/90. Prescribe the next step for his management.
Patient Name:	HT
Gender:	Male
DOB:	7/12/1968
Age:	48
Race:	African American
Insurance:	NHP commercial plan
Medication List:	• Hydrochlorothiazide 25 mg: 1 po qd<br>• Ranitidine 150mg: 1 po bid<br>• Inactive/Past<br>o Lisinopril 40mg: 1 po qd 12/01/2014 -01/01/2015 Discontinued: Allergy - Cough
Problem List:	• Hypertension<br>• GERD<br>*Inactive problems*<br>• Headaches<br>• insomnia
Recent Labs:<br>(DATE for all labs can be 4/2/17)	• A1C: 6<br>• Renal panel:<br>o SCr: 1.3 (range: 0.5-1.1), eGFR 58ml/min, Albumin 4 (3.5-5 G/DL), Calcium 10 (8-10.5mg/DL), CO_2 24 (19-28 mmol/L), chloride 109 (98-110mmol/L), glucose 185 (70-100mg/DL), Potassium 4 (3.1-5.3 mmol/L), Sodium 140 (135-145mmol/L); Urea Nitrogen –BUN- 28 (7-25mg/DL);<br>• Hepatic function panel:<br>o albumin 5 (3.5-5 G/DL); bilirubin total 1.1 (0.3-1.2 MG/DL); bilirubin direct 0.3 (0-0.4mg/DL); alkaline phosphatase total 50 (25-100 U/L); Protein total 7 (6.8-8.6 g/DL); ALT: 10 (range: 9-67 U/L), AST: 15 (range: 13-39 U/L)<br>• Lipid panel: Cholesterol 200 (< 200mg/DL); triglycerides 180 (40-200mg/DL); HDL cholesterol 50 (> 55 mg/DL); LDL cholesterol 130 mg/DL (<130mg/DL)
Recent Vitals:	• Weight: 200 lbs (as of 4/2/2017)<br>• Height:5 ft 10 inches<br>• BMI:28.7<br>• Blood Pressure: 170/96 mm Hg (as of 4/2/2017)
Allergies/Drug Reactions:	• Sulfamethoxazole and Trimethoprim (Hives)<br>• Lisinopril - cough

© 2019 Garabedian PM et al. *JAMA Network Open.*

eTable 6: Diabetes Scenario and Test Patient Data

Scenario/Testing Objectives:	Indication: Type 2 Diabetes<br>• Challenge of prescribing when problem is often accompanied by other problems<br>• Test utility of common/frequent primary care problem<br>• Introduce complexity due to controversy and confusion about second line drugs<br>• Include a kidney disease complication which is especially important because of nuances of recent guideline change.
Scenario Prompt:	A 48 year old male patient, DB, comes in to see you to treat his newly diagnosed type 2 diabetes and he is requesting an oral medication.
Patient Name:	DB
Gender:	Male
DOB:	2/13/1969
Age:	48
Race:	Caucasian
Insurance:	Mass Health
Medication List:	• Lisinopril 40 mg tablet: 1 po qd<br>• Atorvastatin 40 mg tablet: 1 po qd<br>• Aspirin 81 mg tablet: 1 po qd<br>• Metoprolol XL 100mg 1 po qd
Problem List:	• Type 2 Diabetes<br>• Hypertension<br>• Obesity<br>• Hyperlipidemia<br>• Headaches<br>• Heart Failure<br>• IBS<br>*Inactive Problems*<br>• Cough<br>• Depression<br>• Insomnia
Recent Labs:<br>(DATE for all labs can be 4/2/17)	• A1C: 8.2<br>• Renal panel:<br>o SCr: 2.3 (range: 0.5-1.1), eGFR 28ml/min, Albumin 3.3 (3.5-5 G/DL), Calcium 8 (8-10.5mg/DL), CO2 24 (19-28 mmol/L), chloride 109 (98-110mmol/L), glucose 185 (70-100mg/DL), Potassium 4 (3.1-5.3 mmol/L), Sodium 140 (135-145mmol/L); Urea Nitrogen –BUN- 28 (7-25mg/DL);<br>• Hepatic function panel:<br>o albumin 5 (3.5-5 G/DL); bilirubin total 1.1 (0.3-1.2 MG/DL); bilirubin direct 0.3 (0-0.4mg/DL); alkaline phosphatase total 50 (25-100 U/L); Protein total 7 (6.8-8.6 g/DL); ALT: 10 (range: 9-67 U/L), AST: 15 (range: 13-39 U/L)<br>• Lipid panel: Cholesterol 180 (< 200mg/DL); triglycerides 250 (40-200mg/DL); HDL cholesterol 40 (> 55 mg/DL); LDL cholesterol 120 mg/DL (<130mg/DL)
Recent Vitals:	• Weight: 285 lbs (as of 4/2/2017)<br>• Height: 5 ft 10 inches<br>• BMI: 40.9<br>• Last 3 BPs: 138/88 (4/2/2017); 140/82 (4/2/2017); 130/85 (as of 4/2/2017)
Allergies/Drug Reactions:	• Codeine: rash

© 2019 Garabedian PM et al. *JAMA Network Open.*

eTable 7: Restless Leg Syndrome Scenario and Test Patient Data

Scenario/Testing Objectives:	Indication: Restless Leg Syndrome<br>• Test look-alike/sound-alike: ropinirole and risperidone<br>• Include less familiar drug/indication that prescriber might have to look up
Scenario Prompt:	A new patient from Florida, RLS, is a 67 year old woman with restless legs syndrome. She comes to you asking to renew her medication which she recalls was a low dose of risperidone for her restless leg symptoms at night.
Patient Name:	RLS
Gender:	Female
DOB:	8/20/1949
Age:	67
Race:	Unknown
Insurance:	Mass Health
Medication List:	• melatonin 5mg PO QD
Problem List:	• insomnia<br>• restless legs<br>• Obesity
Recent Labs: (DATE for all labs can be 4/2/17)	• Serum ferritin: 80mcg/L
Recent Vitals:	• Weight: 210<br>• Height: 5 ft 5 inches<br>• BMI 34.9 (as of 4/2/2017)
Allergies/Drug Reactions:	• morphine (hives)

© 2019 Garabedian PM et al. *JAMA Network Open.*

eTable 8: Chronic Idiopathic Urticaria ("Itching") and Test Patient Data

Scenario/Testing Objectives:	Indication: Chronic Idiopathic Urticaria<br>• Test look-alike/sound-alike: Hydralazine and Hydroxyzine
Scenario Prompt:	A 35 year old male, IT, comes to his appointment with you requesting hydralazine 25 mg for the itching related to his chronic skin condition, which he said he had used in the past and had worked.
Patient Name:	IT
Gender:	Male
DOB:	1/7/1982
Age:	35
Race:	Unknown
Insurance:	Mass Health
Medication List:	• albuterol inhaler 1-2 puffs q/ 4-6 h PRN SOB<br>• metformin 500mg PO QD
Problem List:	• chronic idiopathic urticaria<br>• asthma<br>• T2DM
Recent Labs:<br>(DATE for all labs can be 4/2/17)	• SCr: 0.95<br>• A1C 9.8
Recent Vitals:	• BP 140/87 (as of 4/2/2017)<br>• Weight: 245<br>• Height: 5 ft 10 inches<br>• BMI 35.2 (as of 4/2/2017)
Allergies/Drug Reactions:	• Diphenhydramine - drowsiness<br>• strawberries, pineapple, onions

© 2019 Garabedian PM et al. *JAMA Network Open.*

eTable 9: Type and Number of Prescribing Errors when Using the Prototype and Vendor during the Usability Testing

	Prototype	Vendors
Total prescribing sessions	128	128
**Error Types**		
Drug prescribed was for treatment of migraine, not prevention	1	3
SIG conflicts with structured data	1	2
Drug Allergy Interaction	1	
Missing azithromycin as part of gonorrhea therapy	1	3
Incorrect dose	1	4
Incorrect frequency	1	2
LASA error	1	3
Drug-drug interaction		4
Incorrect dosing instructions for taper		2
Exceeds max daily dose		1
Incorrect route		6
Missing omeprazole as part of therapy for *H. pylori*		2
Disease Drug Interaction		1
Renal Impairment Contraindication		2
Wrong drug		1
Missing ceftriaxone as part of Gonorrhea therapy		1
Capsule strength not available		1
**Total Errors**	**7/128 (5%)**	**38/128 (30%)**

© 2019 Garabedian PM et al. *JAMA Network Open.*
